# Unusual Structure-Energy Correlations in Intramolecular Diels–Alder Reaction Transition States

**DOI:** 10.3390/molecules191015535

**Published:** 2014-09-29

**Authors:** Justyna M. Żurek, Robert L. Rae, Martin J. Paterson, Magnus W. P. Bebbington

**Affiliations:** Institute of Chemical Science, School of Engineering and Physical Sciences, William Perkin building, Heriot-Watt University, Edinburgh EH14 4AS, UK

**Keywords:** cycloaddition, Diels–Alder, furan, structure-energy correlation

## Abstract

Detailed analysis of calculated data from an experimental/computational study of intramolecular furan Diels–Alder reactions has led to the unusual discovery that the mean contraction of the newly forming C-C σ-bonds from the transition state to the product shows a linear correlation with both reaction Gibbs free energies and reverse energy barriers. There is evidence for a similar correlation in other intramolecular Diels–Alder reactions involving non-aromatic dienes. No such correlation is found for intermolecular Diels–Alder reactions.

## 1. Introduction

The Diels–Alder reaction of furan has been widely used in the synthesis of complex targets and as a probe for the investigation of substituent effects [1,2,3,4,5,6,7,8,9,10,11,12,13,14,15,16,17,18]. We recently reported a comprehensive, computational and experimental study of halogenation effects in intramolecular Diels–Alder reactions [19]. The relative rates of many Diels–Alder reactions can be understood in terms of frontier orbital energy differences between the reaction partners [20,21,22,23,24,25]. Although furan has been widely used as a diene in both inter- and intra-molecular Diels–Alder (IMDAF) processes, and some reaction rates conform to the predictions of FMO theory, substituent effects are less clear cut than for conventional non-aromatic dienes. Other electronic effects, such as positive charge stabilisation, have been shown to be more important than FMO energies in some of these reactions [26,27,28]. Conformational preferences in the tether also play a major role and were the subject of considerable investigation in the 1980s and 1990s [2,29,30].

**Scheme 1:**
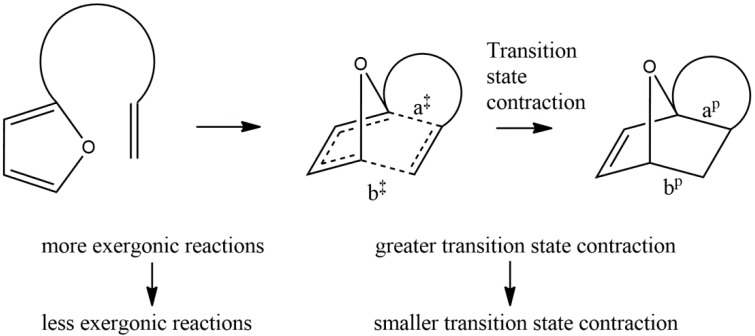
Summary of computational results from the initial set of IMDAF substrates, **1**–**26**.

Our recent work [19] focused on halogenation effects in both diene and dienophile and resulted in the discovery of a C-X dipole-dipole interaction that contributed to the transition state energy. Calculated FMO energies did not correlate well with the experimentally observed reaction rates. Our tentative explanation for this was that the reactions under study had later transition states than are usual for Diels–Alder reactions, such that assuming a reactant-like transition state and using starting material FMO energies was a poor model. Evidence for the character of the transition state was obtained from calculated data, in which those reactions that were predicted to be more exergonic were found to have a greater contraction in the C-C distances in moving along the reaction coordinate from the transition state to the product (and therefore, an earlier transition state), consistent with the Hammond postulate (Scheme 1).

We define the parameter, hereafter referred to as transition state σ-contraction, as follows:

%age TS σ-contraction = 100 * (Δa/a^‡^ + Δb/b^‡^)/2;

where:

Δa = a^‡^ − a^p^ and Δb = b^‡^ − b^p^

This set of reactions are themselves calculated to be much less exergonic than typical Diels–Alder reactions with non-aromatic dienes [28].

## 2. Results and Discussion

While these results were consistent with our original hypothesis that we were studying examples with unusually late transition states, we sought to clarify the matter further and now report the additional analysis of our original data and further high-level calculations that support our initial supposition, as well as uncovering an unusual and, so far as we are aware, unprecedented structure-energy correlation between reaction free energy and the contraction of the C-C distances in going from the transition state to the product.

A conventional method for determining the lateness of a transition state from thermochemical data is to construct a plot of activation Gibbs free energy *vs.* Gibbs free energy of the reaction based on the Leffler Equation (1) [31]:

*∂*∆*G*^‡^ = α *∂*∆*G*°_R_ (1)

**Figure 1:**
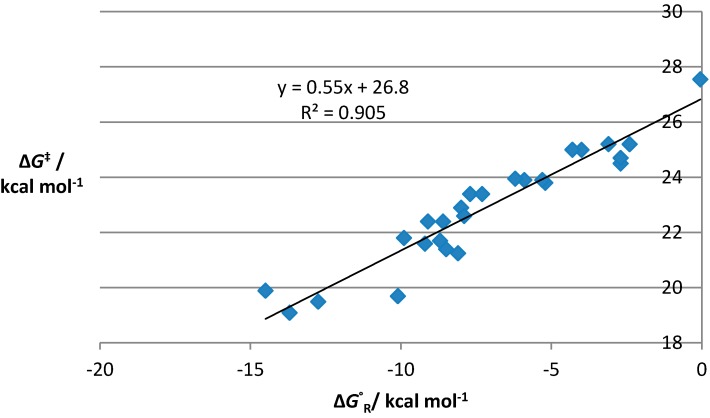
Leffler plot of ∆*G*^‡^ *vs.* ∆*G*^°^_R_ (at 383 K) for our original 26 IMDAF substrates, **1**–**26**.

A plot of ΔG(activation) *vs.* ΔG(reaction) for these systems gives a straight line with gradient α = 0.55, indicating a transition state that is marginally product-like (Figure 1). This is in contrast to related calculations on reactions with conventional dienes, which suggest an enthalpic contribution of about 14% to the activation free energy [32], as well as data from KIE experiments on intermolecular reactions of butadiene and acrolein that also indicate early transition states [33].

**Figure 2:**
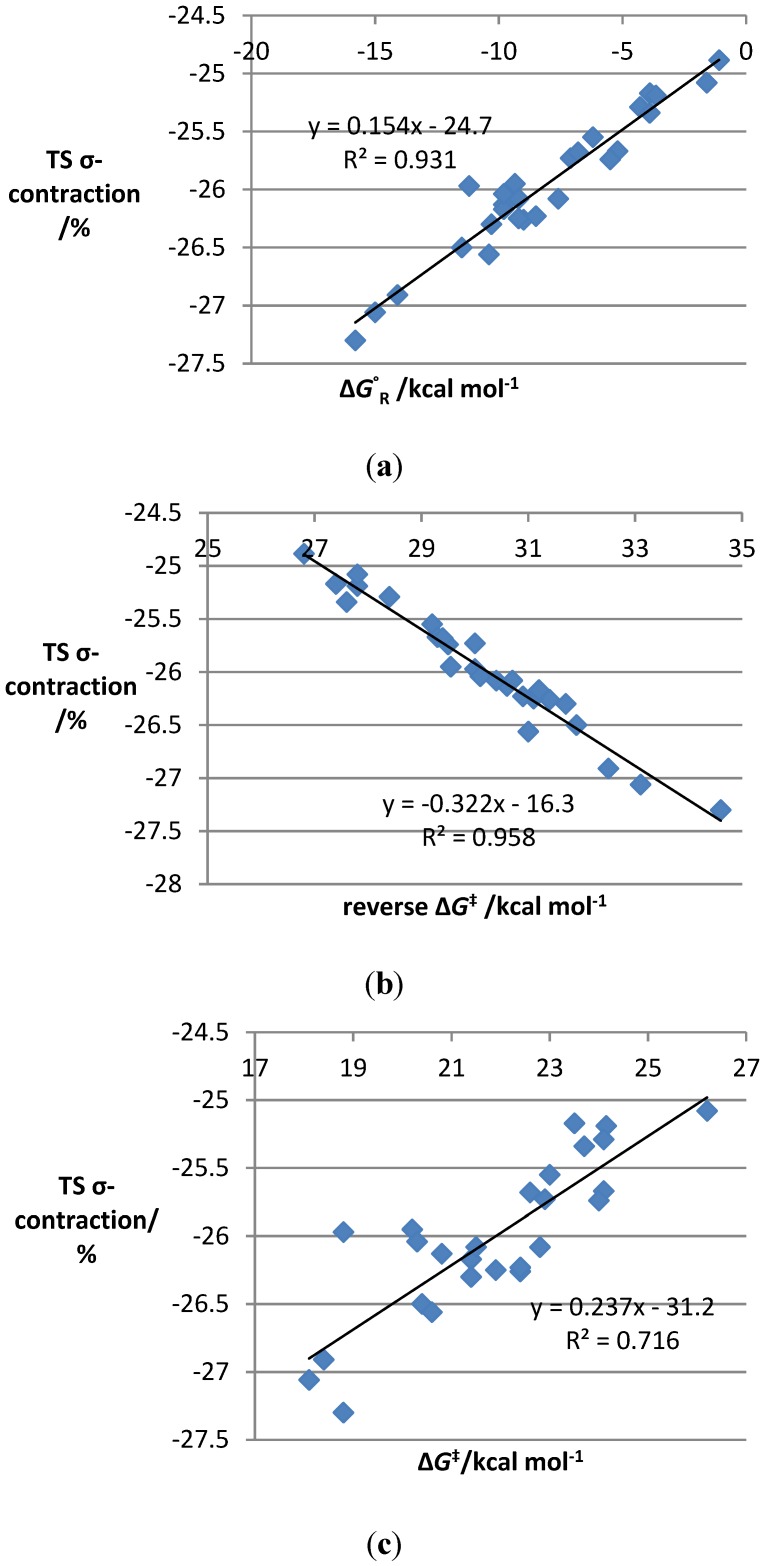
Plots of TS σ-contraction *vs.* key energetic parameters in our experimentally studied data. (**a**) TS σ-contraction *vs.* Gibbs free energies of the reaction for IMDAF reactions **1**–**26**; (**b**) TS σ-contraction *vs.* reverse Gibbs free energy barrier for IMDAF reactions **1**–**26**; (**c**) TS σ-contraction *vs.* Gibbs free energy of activation for IMDAF reactions **1**–**26**.

Examination of the original scatter plots [19] (reproduced in the Supplementary File) reveals a grouping of both activation energies and Gibbs free energies of the reaction according to the type of substrate present, with those containing halogenated dienophiles tending to have higher barriers and less exergonic free energies than those with halogenated furans. Substrates halogenated on both reaction partners were intermediate in both values, and similar trends were observed experimentally. The scatter plot of the average percentage C-C distance contraction from the transition state to the product was strikingly similar to the energy plots, prompting us to probe the nature of a possible correlation between the two. Somewhat to our surprise, a plot of reaction free energy *versus* average percentage C-C bond contraction showed a strong linear correlation (R^2^ = 0.93) (Figure 2a–c).

Perhaps less surprisingly, a linear correlation (R^2^ = 0.96) was also observed between the C-C distance contraction and the free energy change from the transition state to the product (*i.e*., the reverse Gibbs free energy barrier). Correlation with the activation energy was considerably weaker (R^2^ = 0.72). Data points include systems with diverse electronic properties and reaction asynchronicities, so the correlation occurs for a wide range of IMDAF substrates. The gradient of the best-fit line represents the sensitivity of the reaction to changes in substituents.

**Figure 3:**
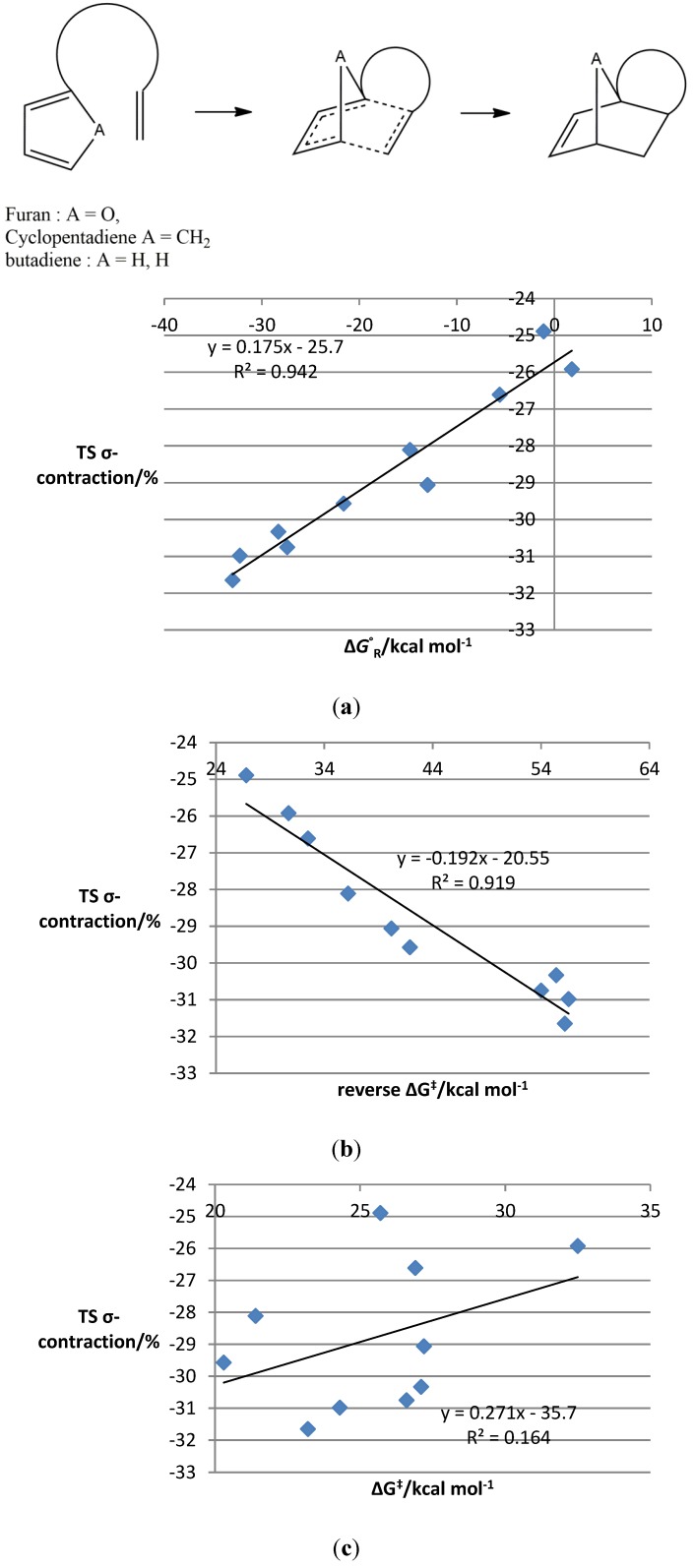
Plots of TS σ-contraction *vs.* key energetic parameters in computed intramolecular DA reactions **37**–**39**, **52**–**54** and **65**–**68**. (**a**) TS σ-contraction *vs.* Gibbs free energies of the reaction for new intramolecular DA reactions **37**–**39**, **52**–**54** and **65**–**68**; (**b**) TS σ-contraction *vs.* reverse Gibbs free energy barrier for new intramolecular DA reactions **37**–**39**, **52**–**54** and **65**–**68**; (**c**) TS σ-contraction *vs.* Gibbs free energy of activation for new intramolecular DA reactions **37**–**39**, **52**–**54** and **65**–**68**.

We next elected to probe the generality of the effect, with related calculations on representative sets of Diels–Alder reactions (see the Supplementary File for the details of the substrates). We chose to examine inter- and intra-molecular reactions of furan, cyclopentadiene and butadiene, including systems related to our original halogenated substrates and also those that exhibited widely varying dienophile electronics with both electron-withdrawing and electron-donating groups. This allowed us to examine whether or not the effects we were observing were confined to a specific set of substrates or to the use of aromatic or cyclic dienes. A combined plot of Gibbs’ free energy of the reaction against transition state contraction for all of the newly modelled intramolecular processes again showed a linear relationship, with a strong R^2^ value of 0.94. This correlation includes substrates with both three- and four-atom tethers, and the correlation appears relatively unaffected by tether length, although this is an issue that would merit further study. Two more plots corresponding to those constructed above showed a similar pattern, with a strong linear correlation between transition state contraction and the activation energy of the reverse reaction (R^2^ = 0.92), but a much weaker one (R^2^ = 0.16) between the contraction and the activation energy of the forward process (Figure 3a–c).

Notably, a Leffler plot using all of the new intramolecular Diels–Alder substrates does not show an obvious straight line. This presumably reflects the difference in the electronic properties of furan, cyclopentadiene and butadiene, which appears to limit our capacity to examine their transition state properties in this way. Nevertheless, a significant structure-energy correlation remains.

**Figure 4:**
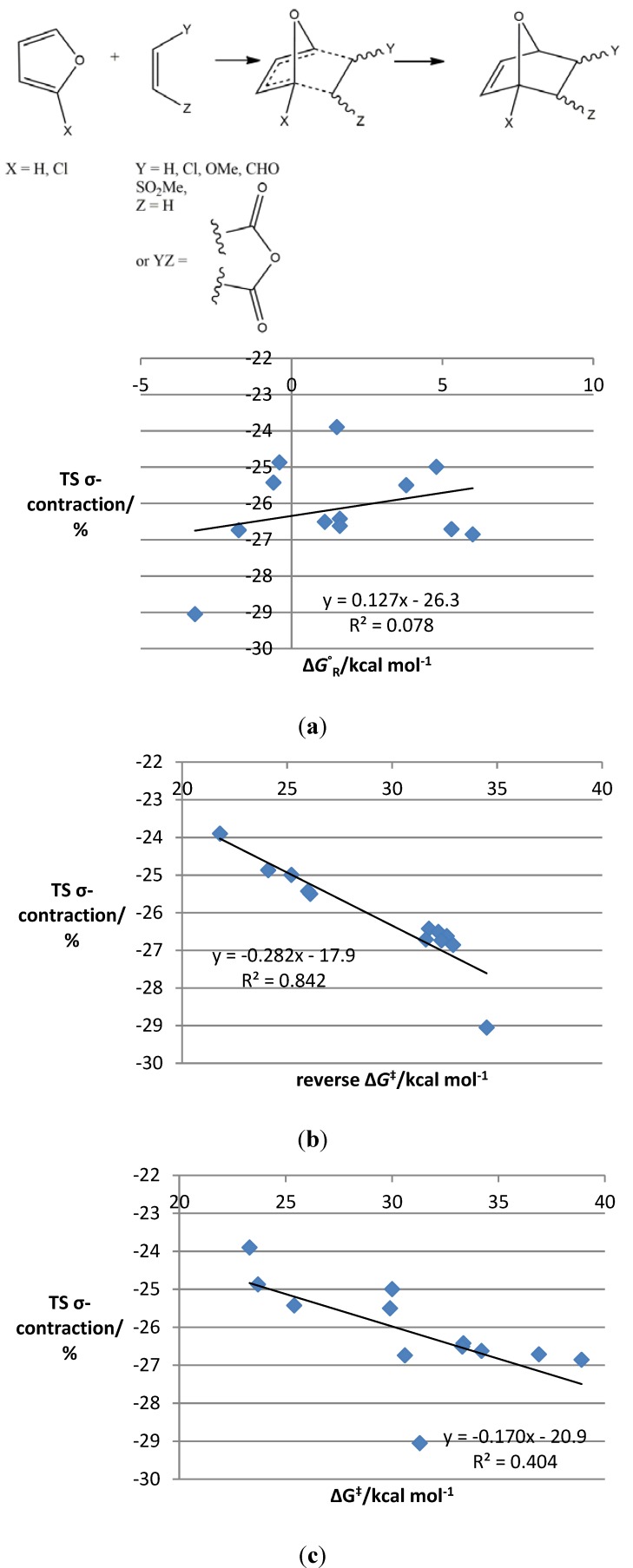
Plots of TS σ-contraction *vs.* key energetic parameters in intermolecular furan Diels–Alder reactions **27**–**36**, **40**–**41**. (**a**) TS σ-contraction *vs.* reverse Gibbs free energy of the reaction for intermolecular furan DA reactions **27**–**36**, **40**–**41**; (**b**) TS σ-contraction *vs.* reverse Gibbs free energy barrier for intermolecular furan DA reactions **27**–**36**, **40**–**41**; (**c**) TS σ-contraction *vs.* Gibbs free energy of activation for intermolecular furan Diels–Alder reactions **27**–**36**, **40**–**41**.

Examination of similar graphs for the intermolecular processes reveals that the patterns observed for the intramolecular reactions were not sustained. Correlations between the key energetic parameters and the transition state contraction were much weaker, for example, for a selection of intermolecular furan DA reactions (Figure 4a–c), although some evidence for the correlation remains for the reverse energy barrier.

The observed correlations were much weaker still for a sample set of intermolecular cyclopentadiene reactions (R^2^ = 0.04–0.6 for reactions **42**–**51** and **55**–**56**) and butadiene reactions (R^2^ = 0.00–0.13 for reactions **57**–**64**), with a range of different dienophiles (see the Supplementary File). The difference in behaviour between the intermolecular and intramolecular reactions is perhaps related to the order imposed by the tethering of the reaction partners in the intramolecular processes. Indeed, the intermolecular processes we have examined appear to have more scope for asynchronicity, which could conceivably lead to a breakdown in the correlation between the energetics and the average value for the contraction of the two new σ-bond distances.

It is apparent from the examination of the calculated transition state data that the correlations we have observed appear to hold for a wide range of different intramolecular Diels–Alder reactions, with very different reaction partners. It is unclear at present whether a simple mathematical expression exists that explains the observed correlations in terms of the underlying intermolecular forces, but in the opinion of the authors, it is likely that the correlation is represented by an approximation to linearity that covers a relatively small range of values for transition state bond contractions. However, the existence of the correlation for this series and, perhaps, as-yet undiscovered reactions may prove useful. For example, such predictable variation of the transition state structure would potentially be of use for those seeking to select reactions for the design of catalytic asymmetric processes, in which the perturbation of the transition state energy and, therefore, structure must be optimised to deliver high enantioselectivity.

## 3. Experimental Section

All calculations were performed with the Gaussian09 program (versions a, c and d) [34]. Geometries of all reactants, products and transition states were initially optimised with DFT theory, the B3LYP functional and the 6–31G(d) basis set. Transition states were obtained via optimisation using the Berny algorithm. The complete basis set (CBS) method of Petersson and co-workers was then used on these geometries to obtain accurate thermochemical data [35,36,37,38]. The specific extrapolation used here is the CBS-QB3 one, the details of which are given in Paragraph 1 of the Supplementary Information. All calculations were performed in the gas phase. Free energies with the CBS-QB3 model were calculated for two temperatures: 298 K and 383 K (the temperature of the experimental studies) for reactions **1**–**26**. There was only a minimal influence of the temperature on the optimised bond lengths for substrates and transition states, and so, the calculations for reactions **27**–**68** were run only at a temperature of 298 K.

## 4. Conclusions

An in-depth analysis of calculated transition state structural data and reaction energetics has revealed an unusual linear correlation between reaction free energy and transition state contraction in intramolecular Diels–Alder reactions. This correlation appears not to be restricted to intermolecular reactions of furan, but does not hold for intermolecular processes. Further investigation is underway to probe the generality of the effect with regard to other types of reaction and to examine its relevance to methodological development.

## Supplementary Materials

Supplementary Figure can be accessed at http://www.mdpi.com/1420-3049/19/10/15535/s1.

## Supplementary Files

Supplementary File 1
