# Supplementary material for: Unusual Structure-Energy Correlations in Intramolecular Diels–Alder Reaction Transition States
Source: Molecules. 2014 Sep 29;19(10):15535–45. doi: 10.3390/molecules191015535 (PMC6272020; doi:10.3390/molecules191015535)
Supplement: Supplementary File 1 [file molecules-19-15535-s001.pdf]

Supplementary Materials

1. Computational Details

CBS Calculations

The complete basis set (CBS) model, namely CBS-QB3, has been used to obtain the thermochemical data of the studied Diels–Alder reactions. Free energies for reactions **1**–**26** were calculated for two temperatures: 298 K and 383 K (the temperature of the experimental studies). Calculations for the remaining reactions **27**–**68** were studied at 298 K. All calculations were performed using the Gaussian09 program [1].

The CBS model employed in this paper uses N^{-1} asymptotic convergence of second-order Moller–Plesset pair energies calculated from pair natural orbital expansions to extrapolate to the CBS limit [2]. The application of CBS extrapolations enables the use of smaller basis sets at second-order, which reduces the calculation time and allows such methods to be used on a wider range of systems. Low levels of theory are used for geometries and vibrational zero-point energies. To obtain a total molecular energy, they are combined with higher-level calculations of the total electronic energy. The CBS-QB3 model consists of the following calculations:

(a) B3LYP/6-311G(*2d,d,p*), geometry optimisation and frequencies;
(b) CCSD(T)/6-31+G(*d'*), energy;
(c) MP4(SDQ)/CBSB4, energy;
(d) MP2/CBSB3, energy and CBS extrapolation (with the G09c, the default number is a minimum of 10 pairs of natural orbitals for this specific basis set used).

Geometries and frequencies in the CBS-QB3 model are obtained with DFT-B3LYP calculations, unlike the original CBS-Q method. The CBS-Q model uses UHF with the 6–31 G basis set for initial geometry and frequency calculations and then MP2(FC) with the same basis set for the final geometry optimisation. Changing to DFT-B3LYP with a larger basis set in the CSB-QB3 method gives more reliable geometries and zero-point energies of stable molecules included in the G2 test set relative to MP2 and gives more consistent structures for transition states [3].

Early work showed that such CBS models would fail in some cases (e.g., some polycyclic systems, perchlorates, *etc.*) [2–4]. As was found later, the reason for this was the use of Mulliken analysis in Pipek and Mezey occupied orbital localisation methods, which, due to the unphysical behaviour in some cases, obtained from the extended basis sets, results in unphysical energy contributions. A new algorithm for localisation was since then employed, in which the populations are measured in a minimal basis (minimum population localisation) and not extended ones. This population method is now implemented in the CBS-QB3 model. It improves its reliability and sorts the previous anomalies caused by the abnormal behaviour of the older population method [4].

The CBS methods are found to be very accurate for thermochemical studies with a mean absolute deviation of around 1.1 kcal/mol for the CBS-QB3 compared to the experimental data on the G2/97 test set [4–6].

2. Substrate Details

Scheme S1. Original data set: depictions of reactions **1**–**26** (corresponding to reactions **a**–**z** in the OBC paper) [7]; scatter plots of Gibbs' free energy of reaction and activation, as well as TS contraction for these reactions.

1

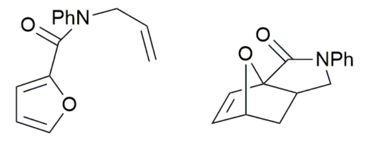

2

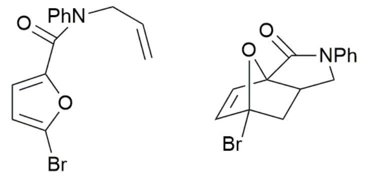

3

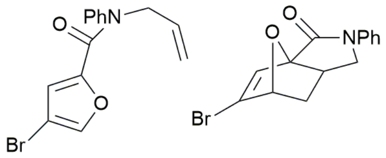

4

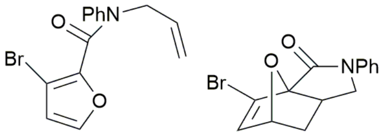

5

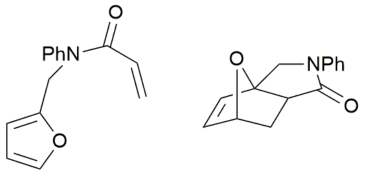

6

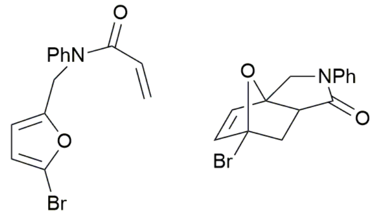

7

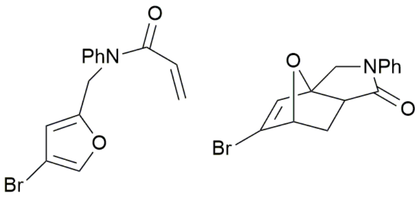

8

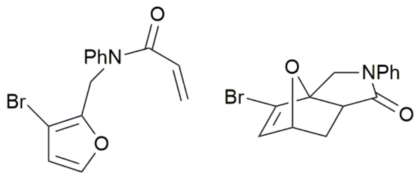

9

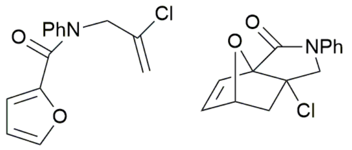

10

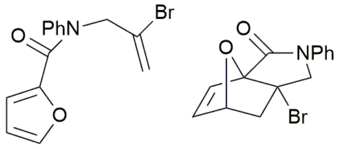

11

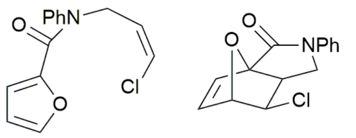

12

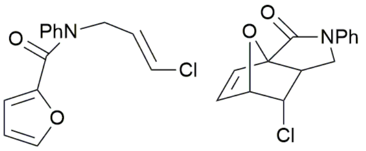

13

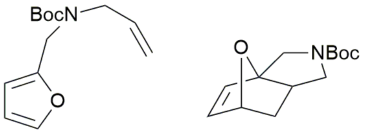

14

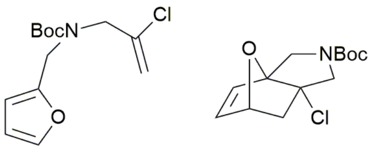

15

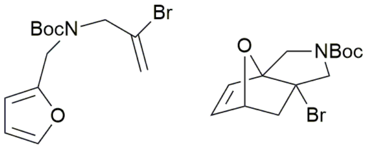

16

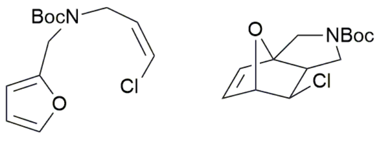

Scheme S1. *Cont.*

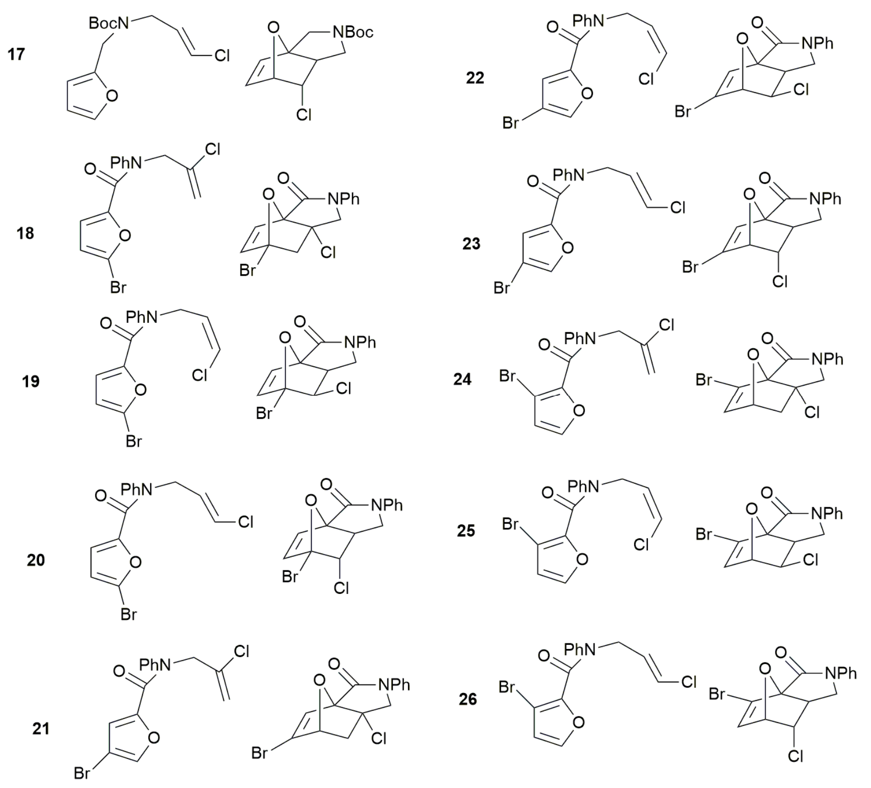

3. Original Scatter Plots of Computational Data from Reactions in the Experimental Study

Figure S1. Scatter plot of Gibbs free energies of the reaction (in kcal·mol^{-1}) for reactions **1–26**. **(**black = unhalogenated; red = halofuran substrate; blue = haloalkene substrate; yellow = dihalogenated substrate).

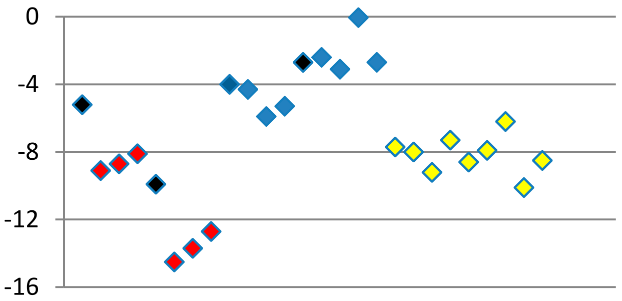

Figure S2. Scatter plot of Gibbs free energies of activation (in kcal·mol^{-1}) for reactions **1–26**.

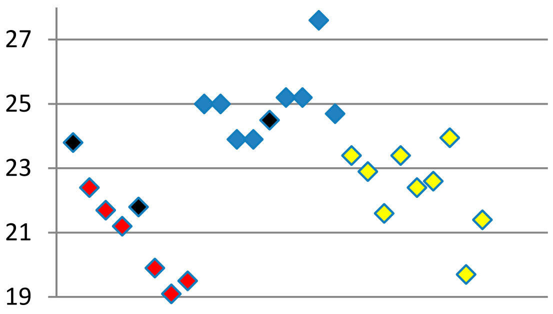

Figure S3. Scatter plot of transition state contraction for IMDAF reactions of **1–26**.

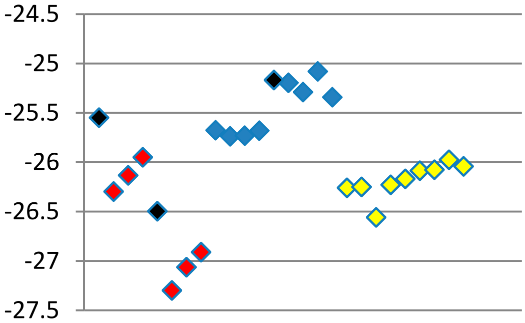

Scheme S2. New Data Sets: Depictions of Reactions of **27–66** and Calculated Data.

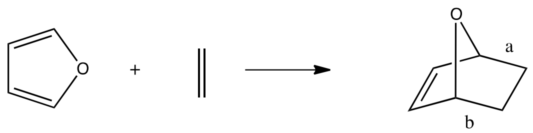

27

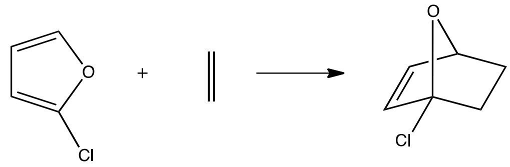

28

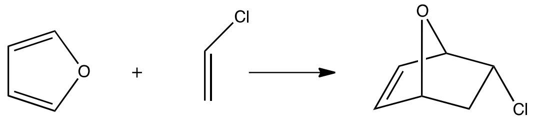

29

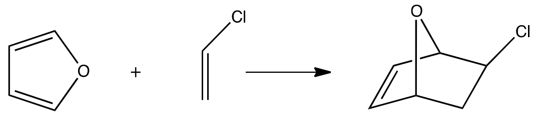

30

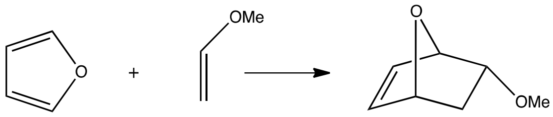

31

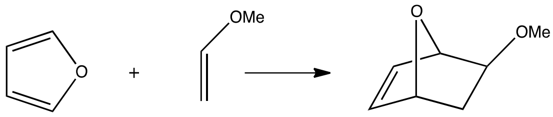

32

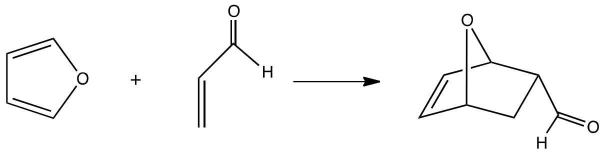

33

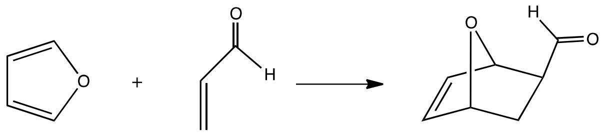

34

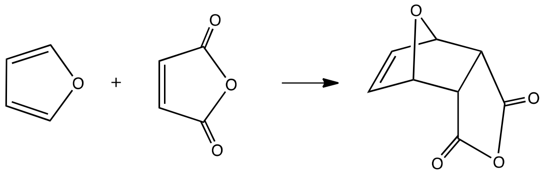

35

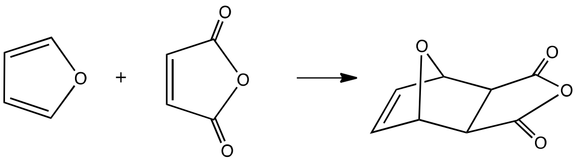

36

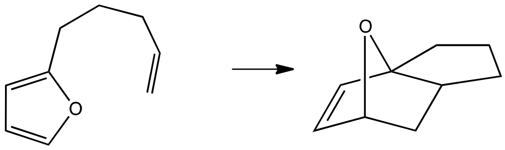

37

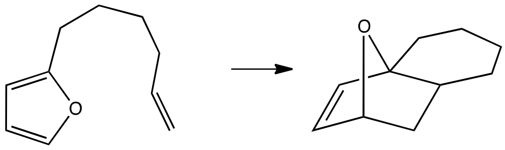

38

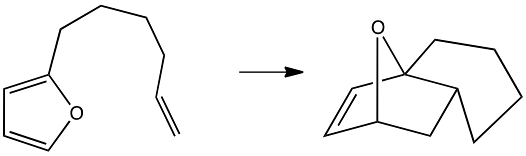

39

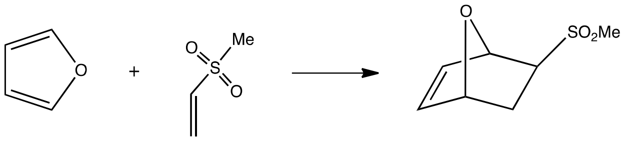

40

Scheme S2. *Cont.*

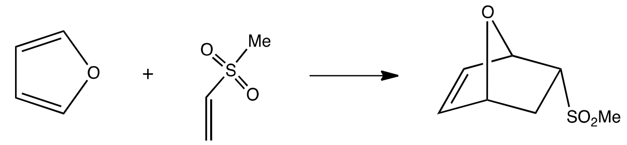

41

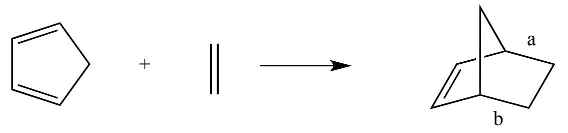

42

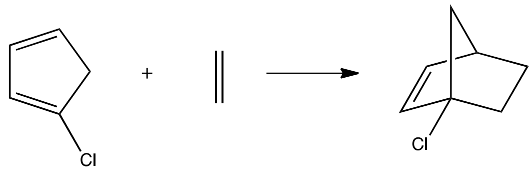

43

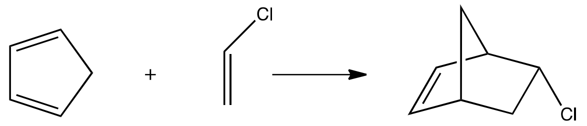

44

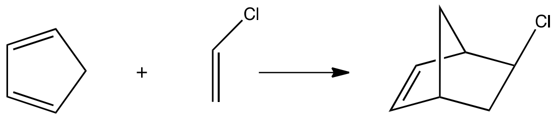

45

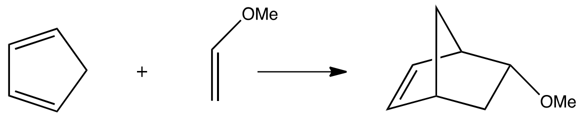

46

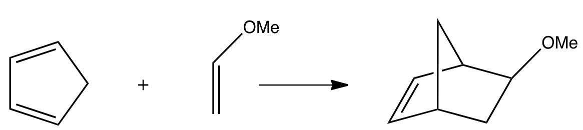

47

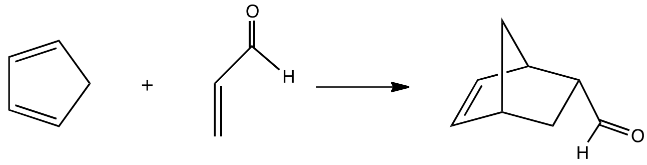

48

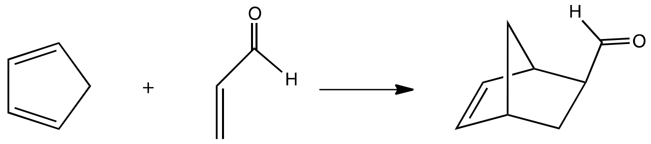

49

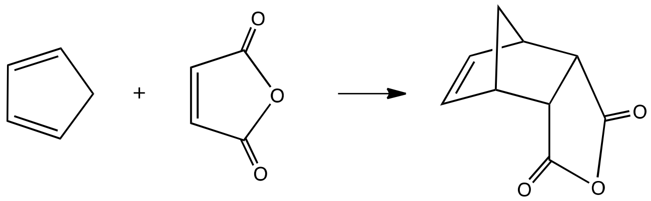

50

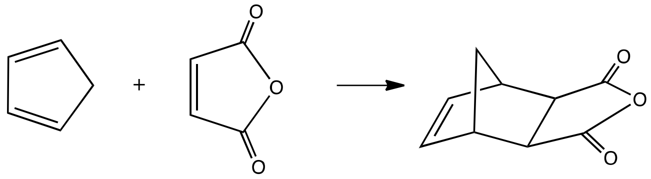

51

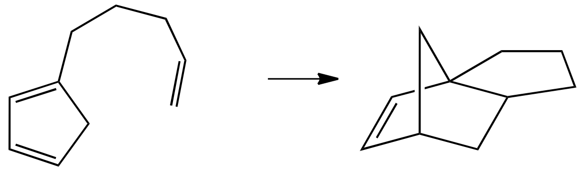

52

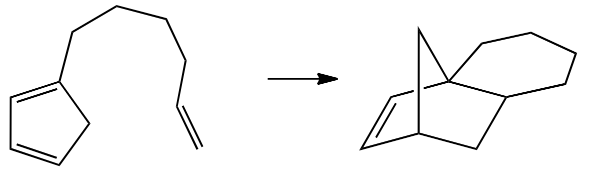

53

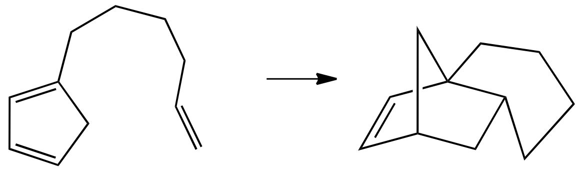

54

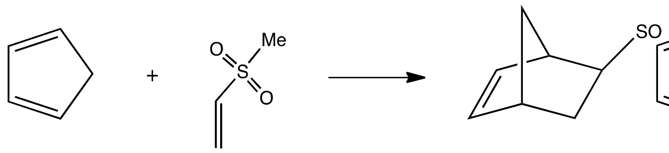

55

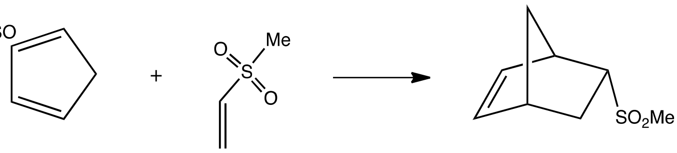

56

Scheme S2. *Cont.*

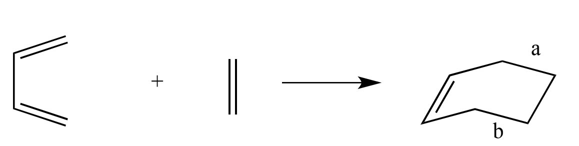

57

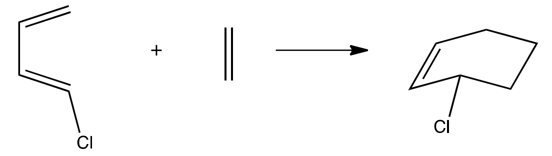

58

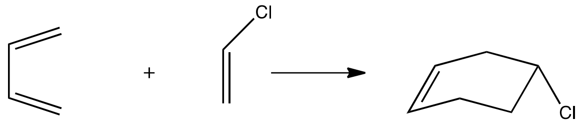

59

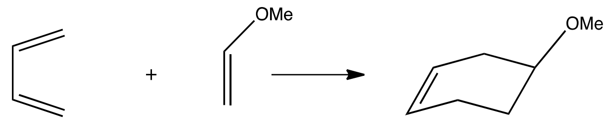

60

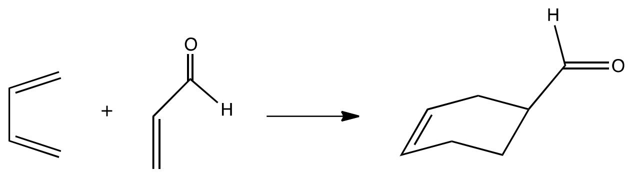

61

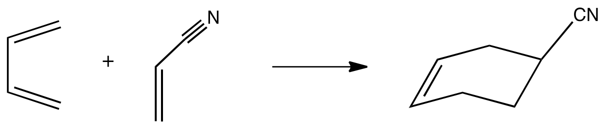

62

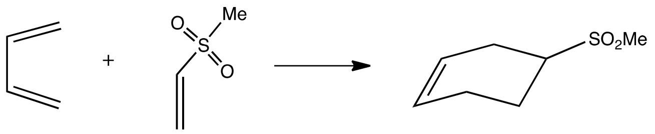

63

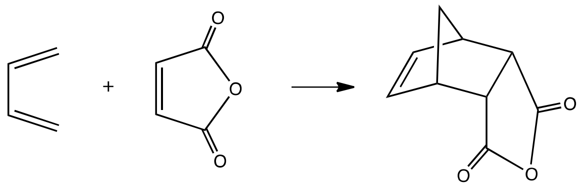

64

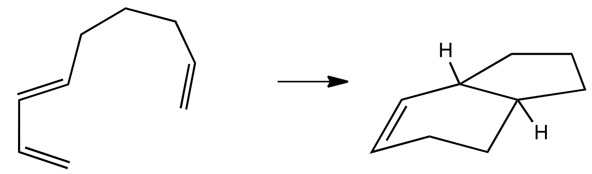

65

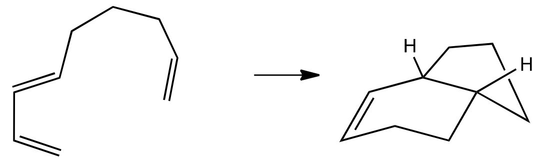

66

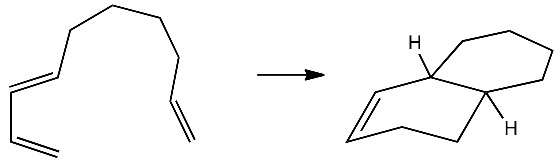

67

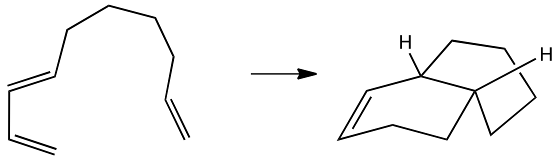

68

Table S1. Table of transition state contraction parameters for reactions **27**–**68**.

Reaction	$\Delta_{act}G°$ kcal/mole	$\Delta_rG°$ kcal/mole	C–C distance $a^‡$	C–C distance $b^‡$	C–C Bond length a^p	C–C Bond length b^p	Δl(a)	Δl(b)	Δl(a)/% from TS	Δl(b)/% from TS	Av%age change from TS
**27**	33.4	1.6	2.128	2.126	1.565	1.565	0.563	0.561	−26.46	−26.39	−26.42
**28**	31.3	−3.2	2.151	2.143	1.524	1.523	0.627	0.621	−29.15	−28.95	−29.05
**29**	33.3	1.1	2.183	2.076	1.564	1.564	0.619	0.512	−28.36	−24.66	−26.51
**30**	34.2	1.6	2.168	2.090	1.562	1.562	0.606	0.529	−27.95	−25.29	−26.62
**31**	36.9	5.3	2.304	1.986	1.563	1.564	0.741	0.423	−32.15	−21.27	−26.71
**32**	38.9	6.0	2.274	2.008	1.560	1.560	0.714	0.448	−31.40	−22.31	−26.85
**33**	30.0	4.8	2.323	1.921	1.589	1.568	0.734	0.354	−31.58	−18.40	−24.99
**34**	29.9	3.8	2.358	1.914	1.586	1.565	0.773	0.349	−32.76	−18.23	−25.50
**35**	23.3	1.5	2.084	2.084	1.586	1.586	0.498	0.498	−23.90	−23.90	−23.90
**36**	23.7	−0.4	2.099	2.099	1.577	1.577	0.522	0.522	−24.87	−24.87	−24.87
**37**	25.7	−1.1	2.142	2.052	1.578	1.571	0.564	0.481	−26.33	−23.44	−24.89
**38**	26.9	−5.6	2.217	2.070	1.580	1.563	0.637	0.507	−28.73	−24.49	−26.61
**39**	32.5	1.8	2.142	2.093	1.572	1.565	0.570	0.528	−26.61	−25.23	−25.92
**40**	30.6	−1.8	2.185	2.092	1.566	1.566	0.619	0.526	−28.33	−25.14	−26.74
**41**	25.4	−0.6	2.231	2.007	1.580	1.572	0.651	0.435	−29.18	−21.67	−25.43
**42**	29.0	−12.3	2.223	2.223	1.568	1.568	0.655	0.655	−29.46	−29.46	−29.46
**43**	27.9	−15.1	2.225	2.233	1.571	1.560	0.654	0.673	−29.39	−30.14	−29.77
**44**	28.9	−13.8	2.159	2.294	1.568	1.558	0.591	0.736	−27.37	−32.08	−29.73
**45**	29.2	−14.4	2.174	2.278	1.564	1.559	0.610	0.719	−28.06	−31.56	−29.81
**46**	33.7	−9.2	2.230	2.200	1.568	1.559	0.663	0.641	−29.71	−29.14	−29.42
**47**	33.0	−9.6	2.061	2.427	1.564	1.559	0.497	0.868	−24.11	−35.76	−29.94
**48**	25.2	−10.0	2.047	2.428	1.569	1.585	0.478	0.843	−23.35	−34.72	−29.04
**49**	25.8	−9.9	2.046	2.455	1.568	1.589	0.478	0.866	−23.36	−35.27	−29.32
**50**	17.5	−15.1	2.197	2.197	1.579	1.579	0.618	0.618	−28.13	−28.13	−28.13
**51**	19.5	−15.4	2.217	2.217	1.577	1.577	0.640	0.640	−28.87	−28.87	−28.87
**52**	21.4	−14.8	2.172	2.210	1.574	1.576	0.598	0.634	−27.53	−28.69	−28.11
**53**	20.3	−21.6	2.159	2.320	1.567	1.585	0.593	0.736	−27.44	−31.70	−29.57
**54**	27.2	−13.0	2.178	2.258	1.571	1.575	0.607	0.683	−27.87	−30.25	−29.06
**55**	22.4	−14.0	2.369	2.115	1.570	1.567	0.800	0.548	−33.75	−25.91	−29.83
**56**	21.8	−15.2	2.335	2.123	1.572	1.573	0.763	0.550	−32.68	−25.91	−29.29
**57**	31.7	−29.0	2.249	2.249	1.536	1.536	0.713	0.713	−31.70	−31.70	−31.70
**58**	30.4	−31.0	2.275	2.213	1.535	1.527	0.740	0.686	−32.53	−31.01	−31.77
**59**	32.1	−31.3	2.288	2.208	1.529	1.534	0.759	0.674	−33.17	−30.53	−31.85
**60**	33.8	−26.0	2.560	2.010	1.532	1.534	1.028	0.476	−40.16	−23.68	−31.92
**61**	27.6	−26.7	2.628	2.019	1.541	1.537	1.087	0.482	−41.36	−23.87	−32.62
**62**	28.4	−29.6	2.519	2.048	1.546	1.534	0.973	0.514	−38.63	−25.10	−31.86
**63**	28.6	−32.7	2.451	2.117	1.534	1.536	0.917	0.581	−37.41	−27.44	−32.43
**64**	21.8	−29.0	2.242	2.243	1.547	1.548	0.695	0.696	−31.00	−31.01	−31.00
**65**	27.1	−28.3	2.179	2.266	1.535	1.561	0.644	0.705	−29.55	−31.11	−30.33
**66**	24.3	−32.3	2.328	2.152	1.555	1.533	0.773	0.619	−33.20	−28.76	−30.98
**67**	26.6	−27.4	2.311	2.171	1.554	1.547	0.757	0.624	−32.76	−28.74	−30.75
**68**	23.2	−33.0	2.293	2.228	1.558	1.532	0.735	0.696	−32.05	−31.24	−31.65

4. Correlation Plots for Intermolecular Cyclopentadiene Reactions 42–51, 55–56

Figure S4. TS σ-contraction *vs*. Gibbs free energies of reaction for intermolecular cyclopentadiene DA reactions.

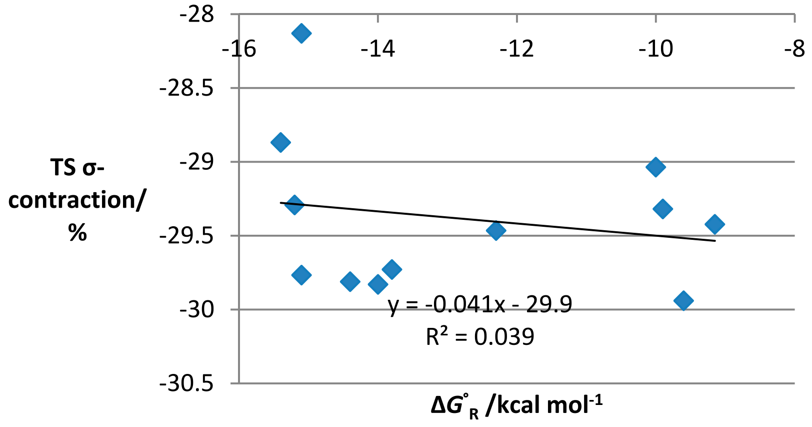

Figure S5. TS σ-contraction *vs*. reverse Gibbs free energy barrier for intermolecular cyclopentadiene DA reactions.

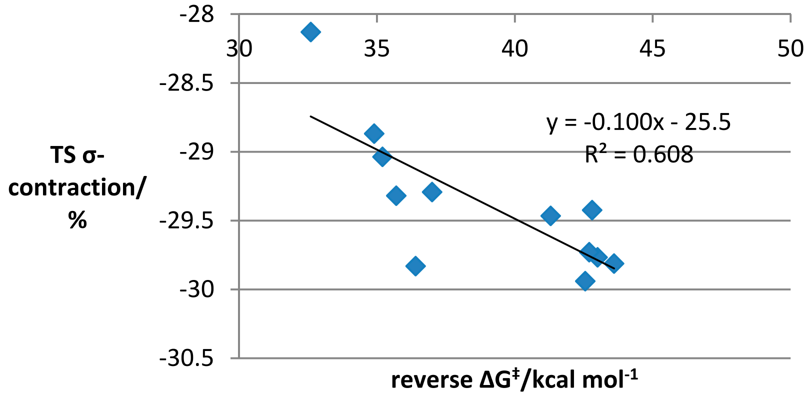

Figure S6. TS σ-contraction *vs*. Gibbs free energy of activation for intermolecular cyclopentadiene reactions.

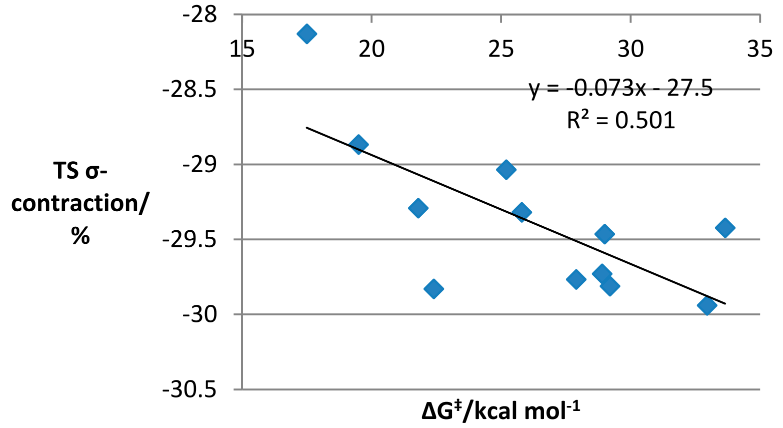

5. Correlation Plots for Intermolecular Butadiene Reactions 57–64

Figure S7. TS σ-contraction *vs*. Gibbs free energies of reaction for intermolecular butadiene reactions.

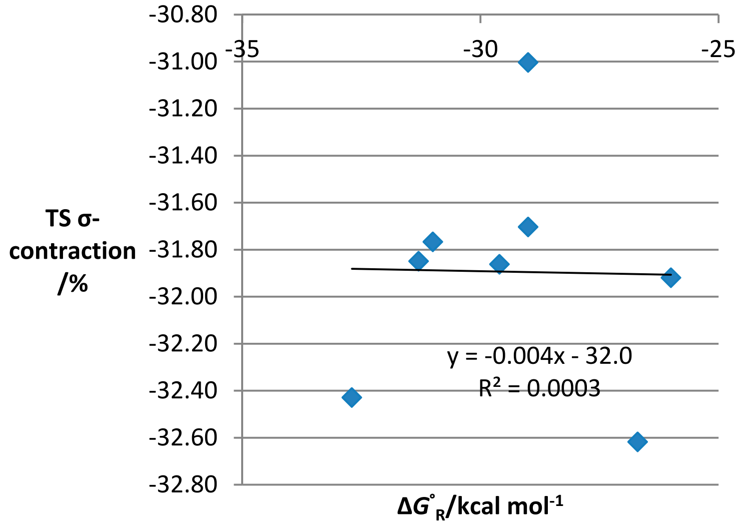

Figure S8. TS σ-contraction *vs*. reverse Gibbs free energy barrier for intermolecular butadiene DA reactions.

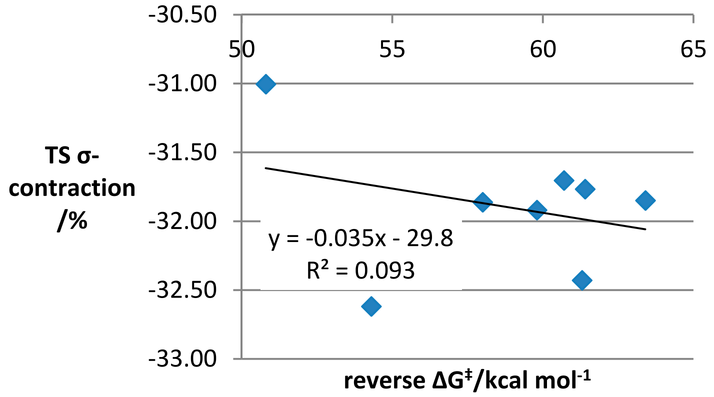

Figure S9. TS σ-contraction *vs*. Gibbs free energy of activation for intermolecular butadiene reactions.

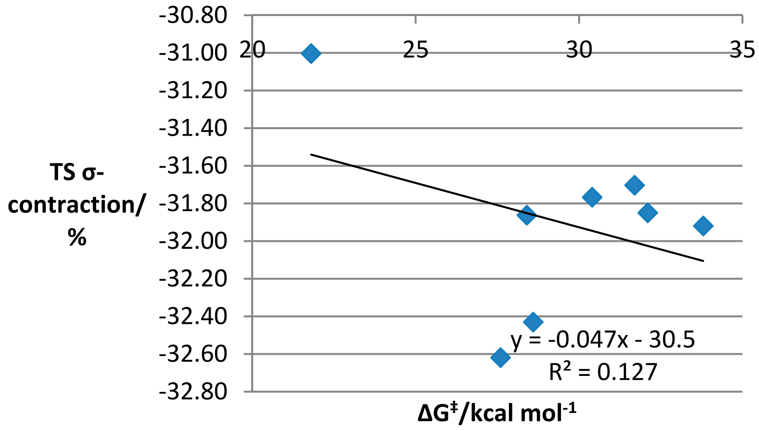

References

1. Frisch, M.J.T.G.W.; Schlegel, H.B.; Scuseria, G.E.; Robb, M.A.; Cheeseman, J.R.; Scalmani, G.; Barone, V.; Mennucci, B.; Petersson, G.A.; Nakatsuji, H.; *et al. Gaussian 09*, Revision D.01; Gaussian, Inc.: Wallingford, CT, USA, 2009.
2. Montgomery, J.A.; Frisch, M.J.; Ochterski, J.W.; Petersson, G.A. A complete basis set model chemistry. VI. Use of density functional geometries and frequencies. *J. Chem. Phys.* **1999**, *110*, 2822–2827.
3. Malick, D.K.; Petersson, G.A.; Montgomery, J.A. Transition states for chemical reactions I. Geometry and classical barrier height. *J. Chem. Phys.* **1998**, *108*, 5704–5713.
4. Montgomery, J.A.; Frisch, M.J.; Ochterski, J.W.; Petersson, G.A. A complete basis set model chemistry. VII. Use of the minimum population localization method. *J. Chem. Phys.* **2000**, *112*, 6532–6542.
5. Curtiss, L.A.; Raghavachari, K.; Redfern, P.C.; Pople, J.A. Assessment of Gaussian-2 and density functional theories for the computation of enthalpies of formation. *J. Chem. Phys.* **1997**, *106*, 1063–1079.
6. Curtiss, L.A.; Redfern, P.C.; Raghavachari, K.; Pople, J.A. Assessment of Gaussian-2 and density functional theories for the computation of ionization potentials and electron affinities. *J. Chem. Phys.* **1998**, *109*, 42–55.
7. Rae, R.L.; Żurek, J.M.; Paterson, M.J.; Bebbington, M.W.P. Halogenation effects in intramolecular furan Diels-Alder reactions: Broad scope synthetic and computational studies. *Org. Biomol. Chem.* **2013**, *11*, 7946–7952.
